# Cellular Intrinsic Mechanism Affecting the Outcome of AML Treated with Ara-C in a Syngeneic Mouse Model

**DOI:** 10.1371/journal.pone.0109198

**Published:** 2014-10-14

**Authors:** Wenjun Zhao, Lirong Wei, Dongming Tan, Guangsong Su, Yanwen Zheng, Chao He, Zhengwei J. Mao, Timothy P. Singleton, Bin Yin

**Affiliations:** 1 Cyrus Tang Hematology Center, Jiangsu Institute of Hematology, the First Affiliated Hospital, Soochow University, Suzhou, Jiangsu Province, PR China; 2 Division of Hematopathology, Department of Laboratory Medicine and Pathology, University of Minnesota Medical Center-Fairview, Minneapolis, Minnesota, United States of America; 3 Department of Laboratory of Medicine and Pathology, University of Minnesota, Minneapolis, Minnesota, United States of America; 4 Thrombosis and Hemostasis Key Lab of the Ministry of Health, Soochow University, Suzhou, Jiangsu Province, PR China; 5 Collaborative Innovation Center of Hematology, Soochow University, Suzhou, Jiangsu Province, PR China; University of Sydney, Australia

## Abstract

The mechanisms underlying acute myeloid leukemia (AML) treatment failure are not clear. Here, we established a mouse model of AML by syngeneic transplantation of BXH-2 derived myeloid leukemic cells and developed an efficacious Ara-C-based regimen for treatment of these mice. We proved that leukemic cell load was correlated with survival. We also demonstrated that the susceptibility of leukemia cells to Ara-C could significantly affect the survival. To examine the molecular alterations in cells with different sensitivity, genome-wide expression of the leukemic cells was profiled, revealing that overall 366 and 212 genes became upregulated or downregulated, respectively, in the resistant cells. Many of these genes are involved in the regulation of cell cycle, cellular proliferation, and apoptosis. Some of them were further validated by quantitative PCR. Interestingly, the Ara-C resistant cells retained the sensitivity to ABT-737, an inhibitor of anti-apoptosis proteins, and treatment with ABT-737 prolonged the life span of mice engrafted with resistant cells. These results suggest that leukemic load and intrinsic cellular resistance can affect the outcome of AML treated with Ara-C. Incorporation of apoptosis inhibitors, such as ABT-737, into traditional cytotoxic regimens merits consideration for the treatment of AML in a subset of patients with resistance to Ara-C. This work provided direct in vivo evidence that leukemic load and intrinsic cellular resistance can affect the outcome of AML treated with Ara-C, suggesting that incorporation of apoptosis inhibitors into traditional cytotoxic regimens merits consideration for the treatment of AML in a subset of patients with resistance to Ara-C.

## Introduction

Acute myeloid leukemia (AML) is an aggressive hematologic malignancy characterized by a clonal expansion of myeloid blasts in the marrow and other sites. Despite the progresses made in AML therapy, such as hematopoietic stem cell transplantation, all-trans retinoic acid [1] and arsenic trioxide [2], the outcomes of most AML patients remain poor. In fact, the 5-year survival for patients with AML diagnosed from 2006 to 2010 was reported to be around 20% [3]. Failures in treatment of AML can be largely attributed to the refractoriness of leukemic cells to current therapies. However, the biological mechanisms underlying leukemic resistance to treatment are unclear.

Mouse models of leukemia have been attractive because of their close mimicking of human diseases in many aspects [4]. The past few years have witnessed a large amount of effort in the search for and the assessment of novel anti-leukemia treatment strategies in these mouse models. Recently, Mulloy’s group evaluated standard cytosine-arabinoside (Ara-C) and doxorubicin regimens in AML xenografts using an immunodeficient mouse model, and showed that this system is useful for evaluation of novel chemotherapy in combination with standard induction treatment [5]. Because the microenvironment plays an important role in leukemic progression and response to therapy [6] and because immune cells are part of the tumor microenvironment [6], we investigated the effects of chemotherapy in immunocompetent mice and attempted to explore the mechanisms for differential drug responses.

Although enormous effort has been put into the exploration of targeted treatment of AML in the recent years, Ara-C remains one of the most effective drugs in the treatment of myeloid malignancies and demands more attentions [7], [8]. It is specific to the S-phase of the cell cycle and therefore exhibits more toxicity to neoplastic cells that are in active synthesis of DNA. However, the outcomes of Ara-C-based treatment vary among patients. Genetic factors of leukemic cells have been associated with their response to treatment. MLL translocations predict poor outcome, whereas other chromosomal abnormalities such as AML1-ETO and inv(16), are associated with better prognosis [9], [10]. Our earlier work demonstrated that Nf1 deficiency conferred Ara-C resistance to AML cells and that leukemic cells with loss-of-function mutation in p53 were selected for and grew out during the acquirement of resistance to Ara-C, indicating these genetic changes affected chemotherapeutic responses of leukemia [11], [12]. Recently, p53 status has also been reported to significantly affect tumor response to targeted therapy [13]. Other factors contributing to the chemotherapeutic response need to be investigated.

To investigate the cellular mechanisms responsible for poor treatment response, we established a syngeneic mouse model of AML by transplanting BXH-2 derived myeloid leukemic cells to immunocompetent mice. The BXH-2 strain of mice spontaneously develops AML at a high incidence, mainly through retrovirally insertional mutagenesis arising from infection by a murine leukemia virus (MuLV) [14] Using this AML mouse model treated with an efficacious Ara-C-based regimen that we developed, we found that leukemic cell load, and the sensitivity of leukemic cells to Ara-C determined the survival. Gene expression profiling was performed to reveal the molecular changes in Ara-C resistant leukemic cells. Of interest, we demonstrated that the Ara-C resistant leukemic cells could be suppressed in vitro and in vivo by inhibition of anti-apoptosis proteins.

## Materials and Methods

### 1. Ethics Statement

The cell line used in this study, B117, was originally established from primary AML cells developed in BXH-2 strain of mice, and published in independent studies thereafter [15]. This cell line is available upon request. All animal work was done in accordance with protocols approved by the Soochow University Institutional Animal Care and Use Committee.

### 2. Cell culture, drug and chemical

B117P and its derived cells were grown as described previously [15]. All culture media and supplements, except noted individually, were obtained from Invitrogen (Carlsbad, CA, USA). Ara-C was purchased from Pfizer Italia S.R.L (Neriviano, Italy). ABT-737 was obtained from Biochempartner Co. (Shanghai, China).

### 3. Animals

C57BL/6J and C3H/HeJ strains of mouse were obtained from Model Animal Research Center of Nanjing, and maintained in a Special Pathogen Free environment in our university animal facility. Six- to eight-week-old B6C3F1 mice generated by mating C57BL/6 and C3H/HeJ were used for leukemia transplantation and drug treatment. Moribund animals were euthanized as the end point in the survival experiments. Since this is an acute leukemia mouse model, these animals did not show obviously detectable sickness until a few hours prior to being moribund. To minimize suffering and difficulty of the mice, the husbandry conditions and health status of mice were monitored on a daily basis for any signs of sickness and maintained from fighting or tail clipping. During the late stage of the experiments, wet food was put on the floor of the cages. Moribund mice were euthanized using a CO_2_ chamber. The criteria for humane euthanasia of mice involved in this study were determined by observation of signs of illness including lethargy, hunched posture, rough coat, and significant body weight loss. For collection of peripheral blood to count cells, mice were bled quickly followed by an immediate application of antibiotic analgesics.

### 4. Cytotoxicity assays

The MTS (3-(4,5-dimethylthiazol-2-yl)-5-(3-carboxymethoxy-phenyl)-2-(4-sulfophenyl)- 2H-tetrazolium, Amresco, USA) tetrazolium assay was used to determine cytotoxic response of AML cell lines, as described before [12]. 1×10^5^ cells were plated into flat-bottom 96-well plates (Corning Inc., Corning, NY, USA) in 200 µL of ASM media with various concentrations of Ara-C. After three days of incubation, 20 µL of MTS solution was added to each well and incubated at 37°C for 2 hours. The optical density at 650 nm was recorded as a reference, and subtracted from OD490 readings to eliminate nonspecific absorbance. Data from individual experiments are presented as the mean percentage of corrected OD490 of triplicate cultures ± SD.

### 5. Histopathology

The various mouse organ specimens were processed for histological analysis according to standard procedure [16]. Briefly, tissues were fixed in 4% paraformaldehyde, paraffin embedded, sectioned, and stained with hematoxylin and eosin. Morphological examination of leukemic cells and peripheral blood from leukemic recipient mice or normal mice used Wright-Giemsa staining method. Images were captured using an Olympus FSX100 at 20X for hematoxylin and eosin stained tissue sections, and 200X for hematoxylin and eosin or 600X for slides stained by Wright-Giemsa, respectively.

### 6. Flow cytometry

B117 cells and bone marrow cells flushed from mouse femurs and tibias were blocked prior to incubation with antibodies staining for Mac-1 and Gr-1 (BD Pharmingen, New Jersey, USA). To detect apoptosis, cells were stained with Annexin V-FITC apoptosis detection kit (Biouniquer, Nanjing, China) following the manufacturer’s protocol. After staining, cells were washed and analyzed on a FACScan (BD Biosciences, Franklin Lakes, USA) using FlowJo software (Tree-Star Inc, Ashland, USA).

### 7. Measurement of B117P cell load using a specific proviral insertion tag

Genomic DNA was extracted from B117P and mouse white blood cells using the TIANamp Genomic DNA Kit (TianGen Co., China), according to the manufacturer’s instructions. B117P specific proviral insertion tags were isolated using APE-PCR, as recently reported [17]. One of the tags was chosen as a molecular marker for B117P cells whose abundance can be reflected by measuring this tag using quantitative real-time polymerase chain reaction (See the following description of qPCR for details). The primers used for amplification of B117 tag were 5′ CTGAGATGAGGCGCAACAT 3′ (forward) and 5′ CCTGTGGTTCAAGTGAAGC 3′ (reverse), and β-actin gene as the internal reference 5′ GATCATTGCTCCTCCTGAGC 3′ (forward) and 5′ GACTCATCGTACTCCTGCTTG 3′ (reverse).

### 8. Gene expression profiling

Total RNA was extracted with TRIzol reagent according to the protocol (Molecular Research Center, Cincinnati, OH, USA), followed by DNase I treatment to eliminate potential genomic DNA contamination. Three samples of B117P and B117HS were analyzed by Super Biotek Co. Briefly, RNA was amplified and labeled using the OneArray Amino Allyl aRNA Amplification Kit (Phalanx Biotech Group, Taiwan) and Cy5 dyes (Amersham Pharmacia, Piscataway, NJ, USA) and hybridized to Mouse Whole Genome OneArray with Phalanx hybridization buffer using Phalanx Hybridization System. Data was normalized using Rosetta Resolver System (Rosetta Biosoftware). A heat map of genes with log_2_ ratio ≥1 or log_2_ ratio ≤−1 and p-value<0.05 was generated using Cluster 3.0 and TreeView and Gene set enrichment analysis was performed using ArrayTrack software (http://edkb.fda.gov/webstart/arraytrack/).

### 9. Quantitative real-time polymerase chain reaction (qPCR)

0.1 µg of total RNA was reverse transcribed with oligo(dT)n, using the SuperScript II First Strand cDNA Synthesis system (Invitrogen) according to the manual. qPCR was performed using SYBR Green method (Applied Biosystems, Foster city, CA, USA). Individual reaction contains diluted cDNA, 400 nM forward and reverse primers each, and 12.5 µL 2x PCR master mixes. PCR reaction was denatured at 95°C for 10 minutes, followed by 40 cycles of 95°C for 15 seconds and 60°C for 1 minute. The fold changes were calculated by the 2^−△△Ct^ method using ABI Prism 7500 SDS Software. The specific primers used for amplification of Dnmt3L were 5′ CTCTGGAAGAGCAATGGCTG 3′ (forward) and 5′ GACTTCGTACCTGATCATCTC 3′ (reverse), Plau 5′ GTGGCAGTGTACTTGGAGCT 3′ (forward) and 5′ GCATCTATCTCACAGTGCTC 3′ (reverse), CD72 5′ GAACAGCGCATCTAACCATCT 3′ (forward) and 5′ GTCGCAGTTGGTTGCTCTG 3′ (reverse), Uba7 5′ GAGTTATACTCCAGGCAGCT 3′ (forward) and 5′ CACTGAGCAGCCAAGTCAG 3′ (reverse), Pdrg1 5′ GAGTGTCTCTGAAGATGTGAT 3′ (forward) and 5′ GTTGACTCCGCAGCCTTTCT 3′ (reverse), and β-actin transcript levels for normalization 5′ CAAGCAGGAGTACGATGAGT 3′ (forward) and 5′ GCCATGCCAATGTTGTCTCT 3′ (reverse), respectively.

### 10. Statistical analysis

Two-tailed Student’s t-tests were performed using Microsoft Excel. A p-value of <0.05 was considered statistically significant. Kaplan-Meier estimates were used to calculate survival, and log-rank was used to calculate the p-value. Survival was defined as from the day of injection of tumor cells.

## Results

### 1. Establishment and characterization of a syngeneically transplanted AML mouse model for evaluation of treatment response

**Figure 1:**
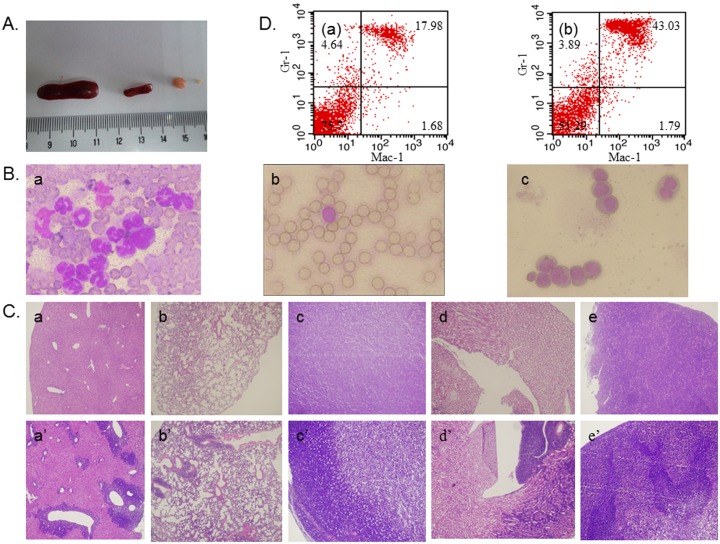
Establishment and characterization of an AML mouse model for evaluating treatment response by transplanting of BXH-2 derived myeloid leukemic cells into syngeneic mice (B6C3F1). Fig. 1A) Gross anatomic examination of spleen and lymph node collected from leukemic (left) or normal (right) mice. Fig. 1B) Morphological examination of peripheral blood smears from (a) recipient mice, (b) normal mice and (c) of B117 cells (Wright-Giemsa staining). Representative fields are shown here. Fig. 1C) Pathologic examination of organ sections, including liver (a & a’), lung (b & b’), lymph node (c & c’), kidney (d & d’), and spleen (e & e’), obtained from normal (a∼e) versus leukemic (a’∼e’) mice (H & E staining). Fig. 1D) Immunophenotypic analysis of bone marrow cells recovered from normal (a) or leukemic (b) mice.

To establish an AML mouse model that is suitable for evaluation of chemotherapeutic response, we intravenously infused 2×10^5^ B117 cells into a syngeneic B6C3F1 mouse. All recipient B6C3F1 mice (n = 12) reproducibly developed a hematologic disease resembling AML around a month following the transplantation, manifested by body weight loss, increased white blood cell counts, and being moribund. The necropsy showed enlarged spleen, lymph nodes and liver (Fig. 1A). Wright-Giemsa stained peripheral blood smears from diseased mice highlighted aggregates of heterogeneous blast cells with prominent nucleoli and nuclear indentations (Fig. 1B). Histopathological studies of liver, lung, lymph node, kidney and spleen revealed diffuse infiltration of the tissues with blast cells (Fig. 1C). Immunophenotypic staining of the bone marrow cells harvested from sick recipient mice showed an increased percentage of Mac-1^+^Gr-1^+^ cells (43%), compared with that for normal mice (18%) (Fig. 1D). These results indicated that an AML mouse model had been successfully created through a syngeneic transplantation of BXH-2 derived leukemia cells.

### 2. Development of an efficacious Ara-C based regimen for the treatment of AML in mice

**Figure 2:**
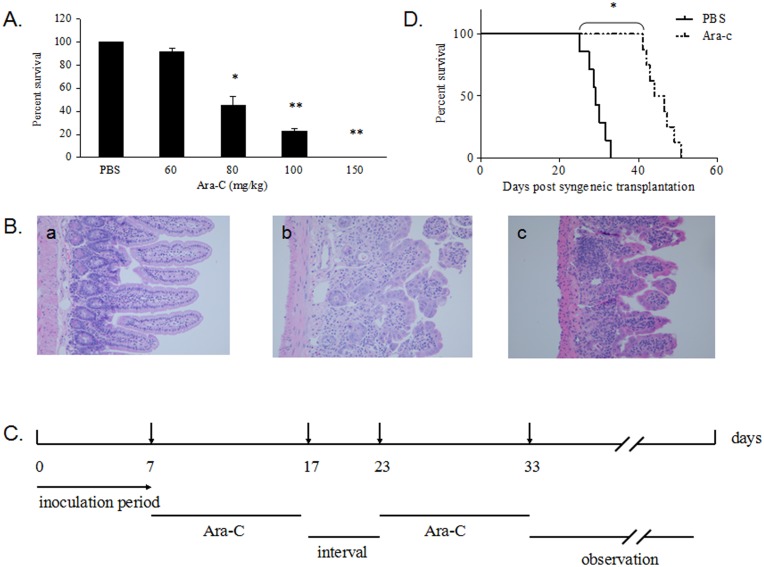
Development of an efficacious Ara-C-based regimen for the treatment of AML in mice. Fig. 2A) The survival rate of mice treated with Ara-C at various doses or with 100 µL of PBS control, in accordance with the treatment scheme described in the text. The mice were watched for a period of 50 days. The results shown here are representative of data obtained from 3 separate experiments. Fig. 2B) Histopathological examination of intestine affects Ara-C treatment. Fig. 2C) Treatment protocol for AML mice. The day of infusion of leukemic cells was considered as day 0. Fig. 2D) Suppression of leukemia using the treatment protocol. B6C3F1 mice transplanted with 2×10^5^ B117P cells via tail vein were treated with either Ara-C (n = 12) or PBS (n = 12) as the control. *p<0.05.

One adverse effect of Ara-C treatment lies in its cause of damage to normal tissues. For the development of a protocol for Ara-C treatment of the mouse AML, it was important to determine the maximum tolerable dose (MTD) of Ara-C that could not only kill the tumor cells in vivo but also spare mice from the damage. To do so, we tested a range of doses of Ara-C on B6C3F1 mice that were intraperitoneally administered once a day for two courses of consecutive ten-day treatments with a five-day interval. We found that injection of 60 mg/kg of Ara-C gave rise to 5%∼10% moribund animals which was further increased as the dose went up, till 100% reaching the end point mostly within the first course at 150 mg/kg (Fig. 2A). During the treatment, a significant body weight loss, a decrease in white blood cell counts, and frequent diarrhea were observed. Histopathological examination of intestine tissues of treated mice was indicative of a damage that became more severe with the increasing doses of Ara-C (Fig. 2B). These symptoms were consistent with the cytotoxicity of Ara-C. Therefore, the dose of 50 mg/kg of Ara-C was chosen for further studies.

Following several rounds of testings, we optimized the treatment scheme as illustrated in Fig. 2C. In order to examine its efficacy, AML mice created by intravenous infusion of 2×10^5^ B117 cells were then treated with the protocol. Compared with the control (PBS) group, Ara-C treatment significantly prolonged the survival of mice for 12∼14 days (Fig. 2D), suggesting that this Ara-C treatment protocol did not kill B6C3F1 mice but effectively suppressed leukemic cell growth in vivo.

### 3. The effect of leukemic load on the outcome of Ara-C treatment

**Figure 3:**
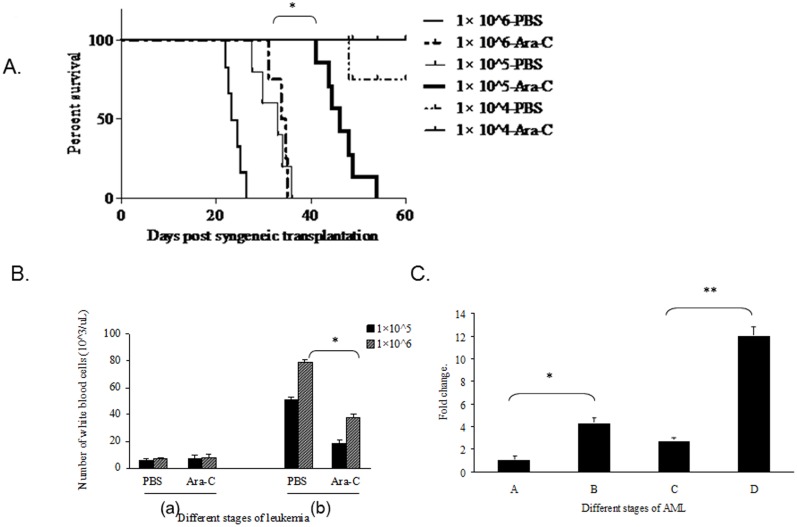
The effect of leukemic load on the survival of recipient mice. Fig. 3A) The survival of B6C3F1 mice receiving different dose of B117P cells and treated with either Ara-C or PBS. Each group included 12 mice. The mice were watched for 60 days. Fig. 3B) The white blood cell counts at different stages of transplanted leukemia in the mouse model. (a) before transplant, (b) after the first course of treatment. Fig. 3C) The abundance of B117 tag in the peripheral blood of recipient mice detected by q-PCR, taking β-actin as the reference, at different time points, (A) 2 days following the transplantation, (B) right before the treatment, (C) following the first course of treatment, (D) following the second course of treatment. *p<0.05, **p<0.01. The data were presented as mean +/− SD of triplicate.

To examine whether AML load had an impact on the treatment outcome, three different numbers of B117P cells were transplanted into B6C3F1 mice, followed by treatment either with PBS as control groups, or with Ara-C according to the above described protocol. As can be seen in Fig. 3A, infusion of 1×10^4^ of B117P cells followed by Ara-C treatment did not cause moribund animal within the period of observation, whereas only 3 (25%) mice died in the PBS control group, indicative of an insufficient induction of reproducible leukemia at this cell dose. When the number of cells increased up to 1×10^5^ and 1×10^6^, the survival of recipient mice became shorter. Consistent with our previous observations, Ara-C treatment delayed the onset of disease, relative to the control groups. Notably, Ara-C treated mice infused with 1×10^5^ B117P cells survived significantly longer than those with 1×10^6^ cells, indicating that a higher level of blast cell load was associated with poor survival of Ara-C treated mice.

The recipient mice, when they became moribund, showed typical leukemic manifestations, with regard to their changed appearance and enlarged hematopoietic organs. The peripheral white blood cells of mice obtained at different stages of transplanted leukemia were counted, and the B117P tag quantified by qPCR. Following the first course of treatment, all four groups of mice displayed high counts of white blood cells, compared to normal B6C3F1 mice (Fig. 3B), consistent with moribund mice from transplanted leukemia and not from Ara-C toxicity. In PBS control groups, mice initially receiving 1×10^6^ B117P cells died gradually, and had a greater increase in the numbers of white blood cells than mice injected with 1×10^5^ cells which were still normal (Fig. 3C). Interestingly, Ara-C treated mice transplanted with either 1×10^5^ or 1×10^6^ cells had decreased cell counts in comparison with respective PBS control mice (Fig. 3B). This is consistent with our observation of the prolonged survival of Ara-C treated recipient mice, indicating, again, that this Ara-C treatment protocol could effectively reduce the number of blast cells. In addition, Ara-C treated mice infused with 1×10^6^ cells exhibited an increased cell counts relative to mice receiving 1×10^5^ cells (Fig. 3B). When they became sick after surviving the second course of treatment, mice showed markedly increased white blood cells (data not shown).

The abundance of B117P cells present in peripheral blood was also monitored by measuring the B117P-specific proviral insertion tag that has previously been identified by APE-PCR [17]. As shown in Fig. 3C, the B117P tag was detected as early as 2 days following the transplantation, and found to be increased later. The levels of B117P tag were decreased following the first course of Ara-C treatment, but became high again when animals were moribund after the second course of treatment. This indicated that changes of the number of B117P cells in the peripheral blood of mice followed a pattern similar to those of white blood cell counts.

The differential survival of mice receiving between 1×10^5^ and 1×10^6^ leukemic cells shown here suggested that leukemic load affected the survival of untreated recipient mice and the outcome of Ara-C therapy for AML.

### 4. Sensitivity of leukemia cells to Ara-C determined the survival of treated AML mice

**Figure 4:**
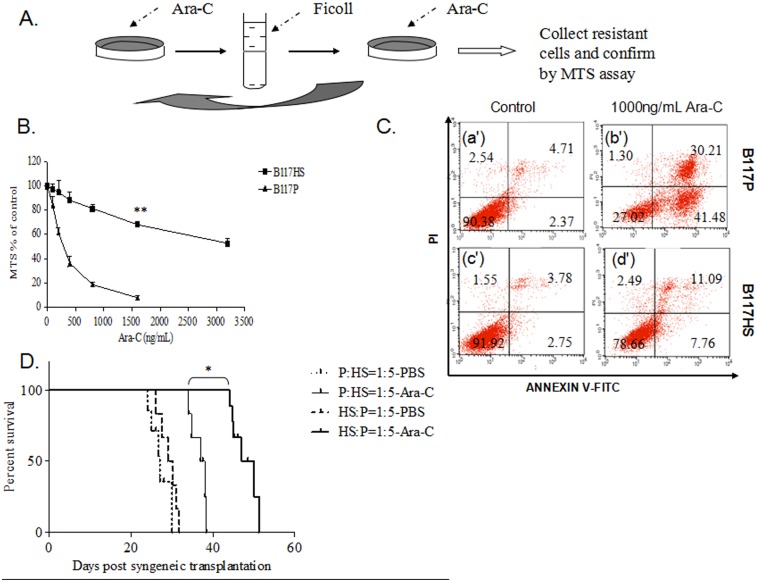
Sensitivity of leukemic cells to Ara-C determined the survival of treated AML mice. Fig. 4A) Selection scheme for generation of Ara-C resistant AML cells (B117HS). Fig. 4B) Comparision of B117P with B117HS in their sensitivities to Ara-C by MTS assay. **p<0.01. Fig. 4C) B117P or B117HS treated either with cell culture medium (a’ and c’) as control or with 1000 ng/mL Ara-C for 48 hours (b’ and d’) were subjected to flow cytometry analysis, according to the Methods. Fig. 4D) Survival of B6C3F1 mice transplanted with 2×10^5^ leukemic cells containing different proportions of B117P and B117HS cells, followed by the treatment with Ara-C or PBS control. Each group included 12 B6C3F1 mice. *p<0.05.

We next asked whether the presence of drug-resistant cells in the leukemic population could impact the outcome of chemotherapy. Firstly, to generate isogenic leukemic cells with different drug sensitivity, we put B117P cells under selection against Ara-C at an initial concentration of 160 ng/mL. This concentration was chosen based on our previous observation that it could give rise to marked cytotoxicity but was no higher than the IC50 of B117P [12]. Thus, over 50% of treated cells could survive this initial treatment (data not shown). As depicted in Fig. 4A, a highly Ara-C resistant cell line, designated as B117HS, was derived from B117P by consecutive exposure to increasing concentrations of Ara-C, as described before [12]. It took about 10 weeks to produce B117HS. To verify the drug resistance of B117HS cells, we then performed MTS assay to determine their sensitivity to Ara-C. As shown in Fig. 4B, the IC50 of B117HS to Ara-C was about ten times higher than that of B117P. Flow cytometry analysis showed that treatment with 1000 ng/mL Ara-C induced apparent apoptosis of B117P and B117HS cells (Fig. 4C).

With the Ara-C resistant cells obtained, we next evaluated the effects of drug sensitivity of leukemic cells on the survival of transplanted animals. To better mimic the clinical context, we mixed B117P and B117HS at the ratio of 1∶5 or 5∶1, and transplanted the mixtures into B6C3F1 mice, prior to the drug treatment. All mice displayed similar signs of illness including lethargy, hunched posture, rough coat, and body weight loss, and eventually being moribund from AML. As shown in Fig. 4D, no obvious difference in the survival of mice between control groups B117P:B117HS (1∶5) and B117P:B117HS (5∶1) was detected. However, Kaplan-Meier analysis showed that, among Ara-C treated mice, B117P:B117HS (5∶1) group survived significantly (p<0.05) longer than B117P:B117HS (1∶5) group, indicating that an increased proportion of B117HS cells resulted in a poor outcome of chemotherapy. These data suggested that the existence of drug-resistant cells in leukemic population could affect the outcome of Ara-C treatment in vivo.

### 5. Gene expression profiling of leukemic cells sensitive or resistant to Ara-C

**Figure 5:**
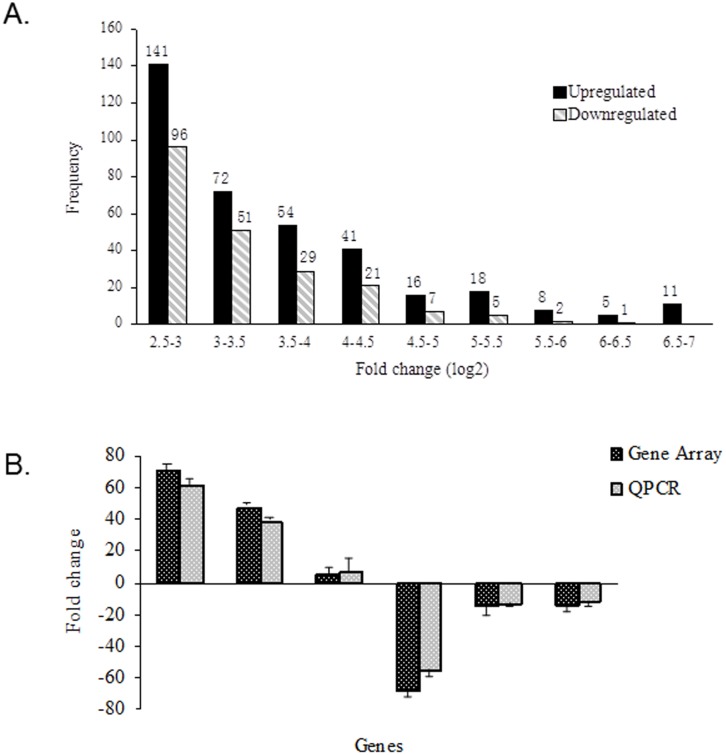
Gene expression profiling of leukemic cells sensitive or resistant to Ara-C. Fig. 5A) Histogram plot shows the distribution of genes with a fold change (log_2_) over 2.5. Fold changes were calculated by Rosetta Resolver 7.2 with error model adjusted by Amersham Pairwise Ration Builder for signal comparison of samples. Fig. 5B) Validation of the differences in expression of Dnmt3L, Plau, Bcl-2, CD72, Uba7 and Pdrg1 genes by quantitative PCR. In order to verify the direction and magnitude of the changes in gene expression induced by resistance of Ara-C in the gene microarrays, q-PCR was performed using primers to the selected genes whose expression levels were significantly altered in the array analysis.

In order to further explore the intrinsic mechanism determining response to chemotherapy at a molecular level, a comparison of gene expression profiles between Ara-C resistant B117HS cells and their parental B117P-derived passage control cells was performed by Super Biotek using Mouse OneArray Microarray. Fluorescent aRNA targets were prepared from 1 or 2.5 µg total RNA samples using OneArray Amino Allyl aRNA Amplification Kit (Phalanx Biotech Group, Taiwan) and Cy5 dyes (Amersham Pharmacia, Piscataway, NJ, USA). Fluorescent targets were hybridized to the Mouse Whole Genome OneArray with Phalanx hybridization buffer using Phalanx Hybridization System. After 16 hrs hybridization at 50°C, non-specific binding targets were washed away by three different washing steps (Wash I 42°C 5 min; Wash II 42°C 5 min, 25°C 5 min; Wash III rinse 20 times), and the slides were dried by centrifugation and scanned by Axon 4000B scanner (Molecular Devices, Sunnyvale, CA, USA). The intensities of each probe were obtained by GenePix 4.1 software (Molecular Devices). Genechip hybridization data were collected, corrected, normalized, and statistically analyzed using programs including statistical Analysis of Microarray, GeneData Analyst, Principal Components Analysis and Cluster. As shown in Fig. 5A, a list of 578 genes with t-test (p<0.01) and fold-change (≥5.5) analysis was obtained, including 366 up-regulated and 266 down-regulated known genes in B117HS cells. Many of these genes were involved in resistance to chemotherapy or tumor formation and metastasis (Table 1). Expression changes for Dnmt3L, Plau, Bcl2, CD72, Uba7 and Pdrg1 genes were confirmed by quantitative real-time PCR analysis using the ABI7500 (Fig. 5B). Our microarray study revealed extensive changes in gene expression levels of Ara-C resistant cells, and some of these may influence chemotherapy response and warrant further exploration.

**Table 1:** Genes upregulated or downregulated in B117HS compared with B117P.

Gene name	Super biotek probe set ID	Gene description	n-fold
**Upregulated**			
Thbs1	PH_mM_0014241	thrombospondin 1	99.99
CD63	PH_mM_0009050	CD63 antigen	99.99
Dnmt3L	PH_mM_0000812	DNA (cytosine-5-)-methyltransferase 3-like	70.09
Plau	mMC007080	plasminogen activator, urokinase	47.5
Tgfb3	mMC024423	transforming growth factor, beta 3	33.15
Rbpms	mMC016216	RNA binding protein gene with multiple splicing	19.03
Zmat3	mMC026312	zinc finger matrin type 3	18.95
ABCB9	mMR028795	ATP-binding cassette, sub-family B (MDR/TAP), member 9	16.46
Vopp1	mMC008041	vesicular, overexpressed in cancer, prosurvival protein 1	12.89
Gdf5	mMC004863	growth differentiation factor 5	11.9
Vegfa	PH_mM_0001624	vascular endothelial growth factor A	10.43
Kdr	PH_mM_0001297	kinase insert domain protein receptor	9.51
Stim1	PH_mM_0014218	stromal interaction molecule 1	8.05
Bcl2	mMR026887	B cell leukemia/lymphoma 2	5.26
**Downregulated**			
CD72	PH_mM_0008460	CD72 antigen	68.63
Psmb9	mMC008677	proteasome (prosome, macropain) subunit, beta type 9 (large multifunc- tional peptidase 2)	46.48
Parp8	mMC009866	poly (ADP-ribose) polymerase famil- y, member 8	33.08
Ripk3	PH_mM_0001581	receptor-interacting serine-threonine kinase 3	23.91
Traf3ip2	mMC006086	TRAF3 interacting protein 2	17.8
SOX4	mMR027907	SRY-box containing gene 4	16.22
Uba7	mMC023220	ubiquitin-like modifier activating enzyme 7	15.13
Pdrg1	mMC022475	p53 and DNA damage regulated 1	14.69
Cmpk2	mMC005508	cytidine monophosphate (UMP-CMP) kinase 2, mitochondrial	9.9
Lgals1	mMC003871	lectin, galactose binding, soluble 1	9.85
Ttc3	mMR026668	tetratricopeptide repeat domain 3	9.04

### 6. Sensitivity of Ara-C resistant leukemic cells to an inhibitor of anti-apoptosis proteins (ABT-737)

**Figure 6:**
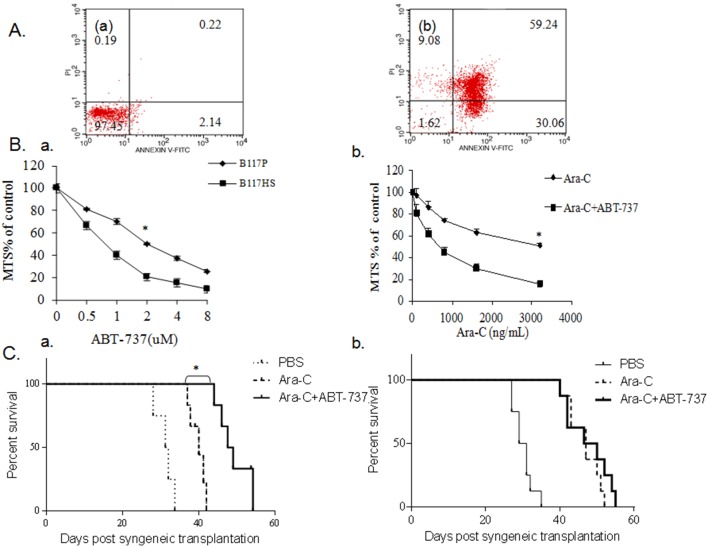
Sensitivity of Ara-C resistant leukemic cells to an inhibitor of anti-apoptosis proteins (ABT-737). Fig. 6A) In vitro induced apoptosis of B117HS cells by ABT-737. B117HS cells were treated with either DMEM medium as control (a) or with 2.0 µM of ABT-737 (b) for two days, followed by apoptosis analysis according to the Methods. Fig. 6B) Sensitivity of B117P and B117HS cells to ABT-737 (a). Cells were treated with 0.5 µM∼8 µM of ABT-737 for three days prior to the MTS assay as described in the Methods. *p<0.05. Sensitivity of B117HS cells to Ara-C alone or in combination with ABT-737. (b). B117HS cells were treated with Ara-C alone or Ara-C combined with 0.4 µM of ABT-737 for three days prior to the MTS assay as described in the Methods. *p<0.05. Fig. 6C) Kaplan-Meier curves of recipient mice treated with Ara-C alone or in combination with ABT-737. B6C3F1 mice were infused with 2×10^5^ B117HS cells (a) or B117P cells (b), followed by the treatment with PBS, Ara-C (50 mg/kg), or Ara-C (50 mg/kg) and ABT-737 (25 mg/kg), respectively. Each group contained 8 mice. *p<0.05.

Because of the result of gene expression profiling including up-regulated Bcl-2, we were curious whether Ara-C resistant cells were responsive to inhibition of anti-apoptotic proteins. Previous studies have shown that ABT-737, a BH3 mimetic, inhibited the pro-survival function of Bcl-2, Bcl-xL and Bcl-w, and induced apoptosis in a variety of cancer cell types [18]–[21]. We first examined the response of B117HS to ABT-737 in vitro. As shown in Fig. 6A, ABT-737 treated B117HS cells exhibited considerable apoptosis and/or necrosis compared to the control. To examine if B117P cells can be effectively treated by ABT-737, we performed MTS assay to measure the sensitivity of B117P to ABT-737. We found that B117P cells were sensitive to ABT-737 treatment, with an estimated IC50 of 2 µM (figure 6B(a)). Interestingly, B117HS exhibited a significantly lower IC50 compared with B117P (p<0.05; figure 6B(a)), indicating that B117HS became even more sensitive to ABT-737 than B117P cells. Then, we determined the sensitivity of cultured B117HS to the combined treatment with Ara-C plus ABT-737 by MTS assay, and found that combined use of 0.4 µM of ABT-737 and an escalation of concentrations of Ara-C ranging from 200 ng/mL to 3200 ng/mL significantly reduced the viability of B117HS cells relative to the treatment with Ara-C alone (Fig. 6B(b)). These data indicated that ABT-737 had a suppressive effect on Ara-C resistant cells, and encouraged us to further examine the in vivo activity of ABT-737. As we observed previously, single agent Ara-C was effective in delaying the progression of leukemia in recipient mice. Excitingly, the combined treatment with Ara-C and ABT-737 suppressed leukemia and prolonged the survival to an even greater extent than the group treated with Ara-C alone (Fig. 6C(a)). However, mice transplanted with B117P-dervied leukaemia had no significantly different survival following the treatment with ABT-737 plus Ara-C compared with Ara-C treatment alone (Fig. 6C(b)). Ara-C resistance of AML cells could be partially reversed by the treatment with ABT-737. These results suggest that inhibition of anti-apoptosis may improve the outcome of Ara-C treatment of AML with intrinsic resistance.

## Discussion

The cure of AML represents a clinical challenge. Although a large body of work has focused on the responses of leukemic cells to a variety of chemotherapeutics, most patients die from relapse. In this report, we investigated the cellular factors contributing to poor prognosis. We found that leukemic cell load within a certain range and intrinsic cellular chemosensitivity are important factors determining the outcome of in vivo treatment of AML.

Targeted therapy of AML with small molecules has been intensely pursued over the past decade. The small compounds act on certain genes or their encoded products that are specifically or preferentially critical to malignant cell survival or proliferation, and can effectively kill tumor cells while having minor side effects. However, the array of molecules that have been developed so far have not proven efficacious in patients, in general [22]. On the other hand, since the standard first line of chemotherapy includes Ara-C we focused on the investigation of AML response to this classic drug.

A variety of mouse models have been developed to study the initiation, progression, and maintenance of AML including chromosome translocation-associated leukemia [23], [24], and mutant gene-induced myeloid malignancy [25]. Recently, by crossing PML-RARA transgenic mice with BXH-2 mice, Kogan’s group has identified a new cooperating pathway to leukemogenesis: Sox4 overexpression accelerated PML-RARα initiated acute promyelocytic leukemia with increased penetrance and reduced latency of disease [26]. Most of the AML mouse models that have been reported are xenografts of human leukemic cells. In contrast to ALL, AML xenotransplantation models are hampered by the problems of inadequate engraftment and a short life span of the animals [27]. Importantly, these systems are usually immunocompromised to facilitate successful engraftment. In this study, we developed an immunocompetent AML mouse model to complement xenotransplant models and mirror the pathology and response to chemotherapy in patients because it is known that the microenvironment can significantly affect tumor response to therapeutics. Based on this mouse model, we developed an Ara-C treatment protocol that has a minimum toxicity but this confirms that suppressed the growth of transplanted AML cells and prolonged the survival of recipient mice. In our study, we found that the two courses of 10-day consecutive treatment scheme with 50 mg/kg of Ara-C worked well.

In clinical practice, the number of leukemic blast cells is associated with the outcome of treatment. Measurement of leukemic load can be a useful tool in order to predict risk of relapse in patients [28]. Tumor load is also an important index for AML treatment by allogeneic bone marrow transplantation (BMT), and intermediate dose of Ara-C is suggested to be used to reduce leukemic load prior to allogeneic BMT [29]. In addition, it is helpful to monitor changes in tumor load following transplantation, since immunotherapy is known to be effective for low tumor burden. Some investigators have found an association between persistence of minimum residual disease and risk of relapse. Nevertheless, data from the literature remain contradictory and further correlations should be established [30]. Using a syngeneic transplantation mouse model, we showed that AML cell load of 1×10^5∼6^ were correlated with the survival of Ara-C treated recipient mice. In agreement, Mulloy observed that early treatment of transplanted AML cells before they expanded out could result in some cures, whereas mice with significant leukemic grafts were not cured using treatment by Ara-C plus doxorubicin [5]. Furthermore, Esteve et al. recently reported that patients with AML harboring a low burden of FLT3-ITD mutation and concomitant NPM1 mutation have a favorable outcome [31]. It appears that heavy leukemic load disrupts normal hematopoiesis more severely or more frequently results in leukemic infiltration of extramedullary organs which is commonly seen in patients with AML. Indeed, we noticed the marked infiltration of transplanted leukemia cells into organs, such as liver, lung, kidney, gastrointestinal tract (data not shown).

It is well established that tumor microenvironment and chronic inflammation play an important role in malignant transformation and progression [6]. Furthermore, it has been found that microenvironment can provide protection from cell death induced by treatment with chemotherapeutics or targeted small molecules for various types of tumors, including solid tumors, multiple myeloma, CLL, CML, AML as well [32]–[34]. Paradoxically, chemotherapy induced damage to tumor niches promote the development of tumor therapy resistance in some instances [35], [36]. Therefore, to evaluate the intrinsic cellular effect on treatment outcome, it would be more appropriate to have a model with a normal microenvironment. Instead of immunodeficient mice, we have established a mouse model with syngeneic transplantation of AML. By following an optimized Ara-C treatment protocol, we demonstrated that the portion of leukemic cells (>1×10^5^) with intrinsic drug resistance affected the survival of treated mice. The intrinsic sensitivity of AML cells affected the outcome of AML treatment with Ara-C. This may help explain the observations that AML cells from different patients showed differential sensitivity to chemotherapy in a xenograft model [5]. Furthermore, heavy tumor load may also give rise to an increased probability of having resistant cell clones or of deriving them during chemotherapy, and influencing leukemia treatment response. Interestingly, our molecular study found extensive changes between sensitive and resistant cells in the expression levels of many genes that are involved in the regulation of several important cellular properties, such as proliferation, survival, metabolism, etc. These results reinforce the idea of intrinsic cellular mechanisms contributing to the treatment outcome of AML. Whether the differentially expressed genes are directly involved in the regulation of leukemic drug response awaits further exploration.

When they become resistant to chemotherapeutics, leukemic cells may retain or even upregulate their intrinsic anti-apoptotic mechanisms. We found that the growth of both Ara-C sensitive and resistant leukemic cells could be inhibited by a Bcl-2-specific BH3 mimetic, called ABT-737. Interestingly, resistant B117HS cells were even more effectively treated by ABT-737 than B117P cells in vitro and in vivo, which may be at least in part explained by the so-called onco-addiction hypothesis. This was a surprise to us, initially, even it was within our conceptual assumptions beforehand, because, although the onco-addiction is an interesting concept which can be translated into efficacious therapy for tumors in some cases, this concept may not equally well suit into every cases. Therefore, based on these analyses, and given the fact that the Ara-C sensitive and resistant B117 cells have similar expression levels of BCL-XL but are different for BCL-2, it seems that the Ara-C resistant B117HS cells are now reliant on BCL-2 function, and that Ara-C resistant leukemic cells with increased BCL-2 expression can become more sensitive to BCL-2 inhibition. ABT-737 has previously been demonstrated to be effective against AML and other forms of hematologic malignancies [18], [19], [21], [37]–[39]. The inhibition of resistant cells by ABT-737 suggests that AML cells with intrinsic resistance to Ara-C remain sensitive to the suppression of anti-apoptotic pathway. There may be potential benefit from using this novel therapy in combination with standard induction Ara-C. In consistence, Jordan and collaborators found that BCL-2 was upregulated in leukemia stem cells enriched primary AML populations, and that BCL-2 inhibitors (ABT-263 or ABT-737) induced cell death by targeting leukemia stem cell mitochondrial energy generation and showed in vivo therapeutic effects against engrafted primary human AML cells [40]. Recently, Abbott Laboratories developed a new Bcl-2-specific BH3 mimetic, ABT-199, which was shown to be efficacious against aggressive Myc-driven murine lymphomas with significantly reduced adverse side effects [41]. Although it seems to be an improved version, whether the new Bcl-2 inhibitory compound can also have in vivo activities against AML cells, especially those with intrinsic chemoresistance, merits further investigations using animal models such as the one that we developed and described in this study.

We have described a syngeneic engraftment mouse model for AML, and showed that this immunocompetent mouse system is valuable for investigating the therapeutic response. The leukemic cell load above certain range and cellular intrinsic chemosensitivity are important factors contributing to the outcome of therapy for AML.
